# Radiomics dataset from chest CT of clinically healthy adults

**DOI:** 10.1016/j.dib.2026.112556

**Published:** 2026-02-09

**Authors:** Viktoria Bedei, Mykola Ostrovskyy, Nilanjan Dey, Taras Kotyk, R. Simon Sherratt

**Affiliations:** a Department of Phthisiology and Pulmonology with a Course in Occupational Diseases, Ivano-Frankivsk National Medical University, Halytska 2, Ivano-Frankivsk 76018, Ukraine; b Division of Pulmonology, Center of Infection Diseases, Ivan Franko 17, Ivano-Frankivsk 76018, Ukraine; c Department of Computer Science & Engineering, Techno International New Town, Kolkata 700156, India; d Department of Human Anatomy, Ivano-Frankivsk National Medical University, Halytska 2, Ivano-Frankivsk 76018, Ukraine; e School of Biomedical Sciences, University of Reading, RG6 6EX, United Kingdom

**Keywords:** Lung, Texture analysis, Imaging biomarkers, Normative reference, Feature extraction, PyRadiomics, Quantitative imaging

## Abstract

This data note describes a structured dataset of lung radiomic features derived from thoracic noncontrast computed tomography examinations of 100 subjects (47 males, 53 females; aged 15–74 years). Participants were selected on the basis of the absence of known lung, pleura, and mediastinum diseases in clinical records and radiology reports, as well as systemic diseases affecting the respiratory system. The included computed tomography studies were performed on a single multidetector CT scanner (Siemens Healthineers SOMATOM go. Now), using a uniform protocol (110 kVp; reconstructed slice thickness 0.8 mm; Br60-type lung kernel).

For each case, the target thin-slice DICOM series was converted to the NIfTI format. The lung lobes (“raw” masks), vessels and air pathways were segmented automatically with TotalSegmentator. In addition to “raw” lobe masks, vessel/airway-subtracted (parenchyma) masks were generated. Lobe masks (left lung – 2, right lung – 3) were also combined into the left lung, right lung, and both lungs, resulting in eight ROIs per subject for each mask type – with (“raw”) and without vessel/air pathways (“parenchyma”).

For each SubjectID×ROI, radiomic features (107 “original” features – shape, first-order, and texture families) were extracted via a PyRadiomics-based pipeline with fixed settings (B-spline interpolation; resampling to 1 × 1 × 1 mm; bin width 25 HU; absolute resegmentation) in two attenuation ranges: −1000 to +200 HU and −950 to 0 HU. The dataset is distributed as (i) a CT protocol table, (ii-iii) two feature tables (“raw” and parenchyma masks), (iv) a JSON file with a computational environment description, (v) a Python extraction script, and (vi) a dictionary file.

This dataset can serve as a normative reference for lung radiomics, a benchmark for harmonization and robustness studies, and a control cohort for comparative modelling in diffuse lung diseases, as well as region-specific diseases requiring lobar or single-lung-specific radiomic features (such as emphysema, chronic obstructive pulmonary disease, Swyer–James–MacLeod syndrome, asbestosis, and silicosis).

## Graphical abstract

**Figure:**
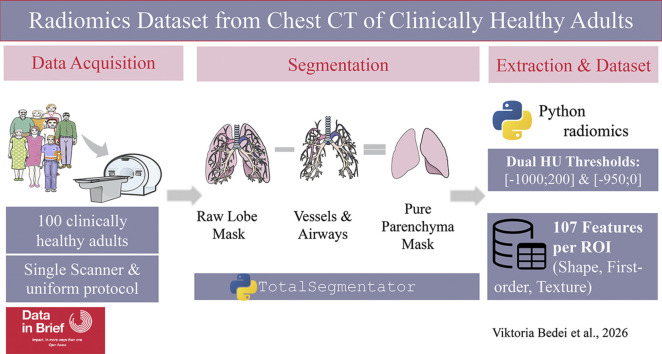

Specifications Table

**Table:** 

Subject	Health Sciences, Medical Sciences & Pharmacology
Specific subject area	CT-based lung radiomics in radiologically normal adults
Type of data	Tabular data (CSV); JSON metadata; Python-script.
Data collection	Participants without pulmonary pathology and other pulmonary-associated systemic diseases, who underwent native chest CT examinations with further radiological evaluation confirming the absence of pulmonary pathology, were included in the dataset. Data were collected with Siemens Healthineers SOMATOM go.Now scanner (110 kVp, 0.8 mm slice thickness, Series Description: Thorax 0.80 Br60 S5). DICOM series were converted to NIfTI (dcm2niix). Lung lobes, pulmonary vessels, and tracheobronchial tree were segmented using TotalSegmentator. Radiomics features were computed with PyRadiomics (resampling – 1 × 1× 1, binWidth – 25)*.*
Data source location	Institution: Center of Infection Diseases; Ivano-Frankivsk National Medical University Country/Region: Ivano-Frankivsk, Ukraine Imaging device: Siemens Healthineers SOMATOM go.Now
Data accessibility	Repository name: Mendeley Data Data identification number: doi: 10.17632/8mdb9xfbnx.1 Direct URL to data: https://data.mendeley.com/datasets/8mdb9xfbnx/1
Related research article	None

## 1 Value of the Data

• This dataset provides lung radiomic features derived from CT examinations of 100 clinically healthy subjects obtained with a single-scanner/uniform protocol (fixed kVp, slice thickness, reconstruction kernel), enabling analyses with minimal acquisition heterogeneity.
• The dataset includes eight anatomically defined ROIs per subject (five lobes + left lung + right lung + both lungs) and two fixed HU resegmentation ranges, supporting region- and window-specific sensitivity analyses.
• The dataset can be used as a feature-level reference cohort in studies on diffuse lung diseases (e.g., sarcoidosis, interstitial lung disease, and COVID-19 changes) to quantify and report deviations of patient radiomic features from a controlled “normal” distribution when extracted via matched settings.
• In longitudinal research designs, the provided reference distributions can be used to contextualize within-subject changes in radiomic features by comparing observed changes against expected variability in nonpathological lungs under a stable protocol.
• The availability of lobe- and lung-level ROIs with/without vessels and airways enables ROI-specific comparisons and sensitivity analyses in studies focusing on region-dependent patterns (e.g., tuberculosis, oncology, emphysema, COPD, Swyer‒James‒MacLeod syndrome, asbestosis, and silicosis), using matched extraction settings.

## 2 Background

Radiomics workflows for chest CT are commonly composed of image conversion, segmentation, and feature computation steps; the lack of standardized algorithm definitions and image processing has been reported to hamper reproducibility and comparability [1]. The lack of standardized definitions and validated reference values has been described as a barrier to clinical use, whereas standardization has enabled verification and calibration across software implementations [2]. The robustness of radiomic features in physiological tissue has been described as a prerequisite for quantitative imaging applications [3]. Public thoracic CT resources are often related to specific diseases (e.g., nodules, cancer, embolism, COVID-19, community-acquired pneumonia, and tuberculosis/nontuberculous mycobacterial pulmonary disease) [[4], [5], [6], [7], [8], [9], [10]]. Therefore, reference feature distributions from CT examinations interpreted as normal are needed to contextualize whether deviations observed in disease cohorts represent meaningful departures from expected “normal” variability or instead reflect methodological and acquisition-related variability. To address this, a single-scanner cohort of noncontrast thoracic CT images interpreted as having no clinically relevant thoracic abnormalities was assembled; automated segmentation was performed with TotalSegmentator [11], and PyRadiomics features [1] were computed via fixed settings across subjects, ROIs, and two HU resegmentation ranges to improve acquisition/processing homogeneity. Moreover, lobe and whole-lung features in two fixed HU intensity ranges are provided, as they extend applicability reuse to capture spatial heterogeneity [12] along with the influence of the voxel intensity range (“intensity window”) on radiomic feature values [13,14].

## 3 Data Description

**Fig. 1:**
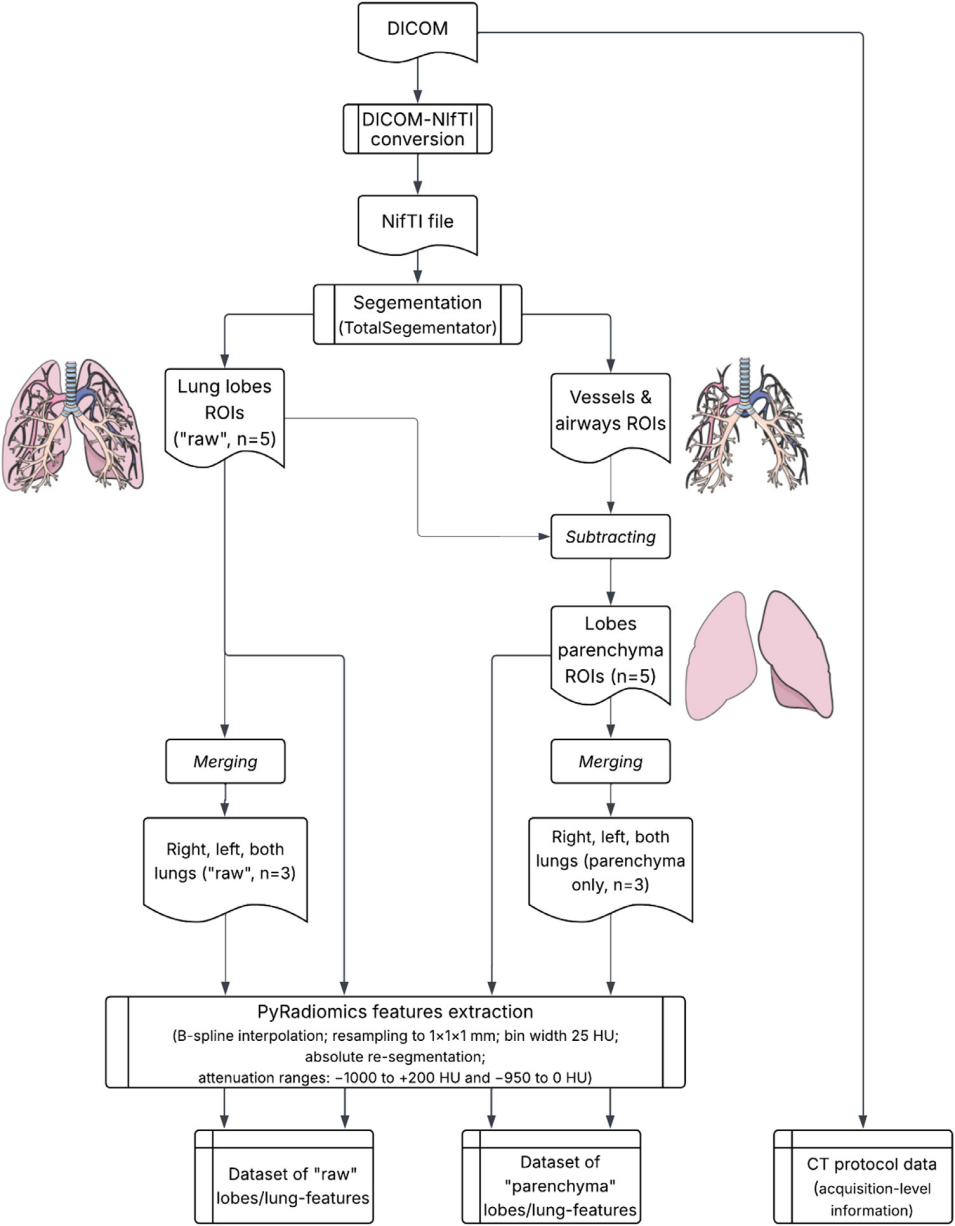
Data extraction workflow. A thoracic CT series was converted from DICOM to NIfTI, while acquisition parameters were recorded in ct_protocol.csv. Segmentation of the NIfTI volume with TotalSegmentator yielded: (i) five lung-lobe ROIs (“raw”) and (ii) pulmonary vessel and airway ROIs. Parenchyma-only ROIs were generated by subtracting (ii) vessel/airway masks from (i) each lobe. Then, lobes were merged into “raw” and “parenchyma-only” lung ROIs, respectively (left, right, and both-lung ROIs), yielding 16 ROIs per subject. PyRadiomics features were extracted for each ROI in two attenuation (HU re-segmentation) ranges (−1000 to 200 HU and −950 to 0 HU), and stored in radiomics_raw.csv and radiomics_parenchyma.csv according to ROI type. A Python script with implemented workflow logic and feature extraction settings is provided in the dataset.

The dataset is distributed as six files linked with a single key (SubjectID, n = 100). Details of CT acquisition are provided in ct_protocol.csv (one row per subject). Two feature tables (radiomics_raw.csv and radiomics_parenchyma.csv) contain basic demographic data (age, sex), radiomics features extracted under identical settings for 8 ROIs (5 lobes; right, left, and both lungs) and 2 HU resegmentation ranges. The difference between the two feature tables is the ROI definition: radiomics_raw.csv uses masks including pulmonary vessels and central airways, whereas radiomics_parenchyma.csv uses parenchyma masks obtained by subtracting vessel and airway labels prior to feature extraction (Fig. 1). The data dictionary file describes the core variables for reuse. The computational environment and the extraction script used are provided in JSON-format and Python-script, respectively (Table 1).

**Table 1:** Overview of the dataset.

File	Format	Unit/size	Key content	Linkage/notes
ct_protocol	csv	100 rows (1/subject)	Scanner and protocol descriptors (e.g., Manufacturer/Model, kVp, SliceThickness, CTDIvol, TubeCurrent, ExposureTime)	Primary key: SubjectID
radiomics_ raw	csv	1,600 rows (100 × 8 × 2)	Subject-level demographics (Age, Sex), ROI and HUWindow labels, PyRadiomics “original_*” features	SubjectID links to ct_protocol.csv; ROIs include vessels/airways
radiomics_ parenchyma	csv	1,600 rows (100 × 8 × 2)	Same structure as radiomics_raw	Identical extraction settings; ROIs exclude vessels/airways via subtraction
data_dictionary	csv	14 rows	Variable definitions (name, type, allowed values/units, notes)	Covers ct_protocol and radiomics_*
radiomics_ extractor	py	1 file	Feature extraction script used to generate the CSV tables	Provides exact configuration used
radiomics_meta	json	Key-value	Software/hardware environment (OS, Python, TotalSegmentator, PyRadiomics, CUDA/GPU and library versions)	Supports reproducibility of the pipeline

## 4 Experimental Design, Materials and Methods

### 4.1 Study population

Individuals who underwent chest CT for various clinical indications or routine check-ups were retrospectively screened. Examinations interpreted as having no clinically relevant abnormalities in the lung parenchyma, pleura, or mediastinum were considered potential controls.

Inclusion criteria:

• Subjects without clinically relevant respiratory pathology, significant clinical records and radiology reports regarding lung diseases, as well as systemic diseases affecting the respiratory system.
• Native (non-contrast) thoracic CT, including the entire lungs, was acquired on the designated scanner (Kernel Br60, slice thickness 0.8 mm).
• Availability of the standard thin-slice series with reconstruction kernel Br60 (Series Description: Br60 S5) imaging.

Exclusion criteria:

• CT examinations with any reported pulmonary, pleural, or mediastinal pathology.
• Prior lung resection or obvious anatomical distortions.
• Motion artefacts or technical issues that compromise segmentation.

A minimum sample of 100 analysable chest CT examinations to obtain stable estimates of radiomic feature distributions under a single, tightly controlled acquisition protocol was planned. Data collection was therefore stopped once 100 eligible adult subjects had accrued (47 males, 53 females; mean age ≈ 43 years).

### 4.2 CT acquisition protocol

All included CT scans were performed on a single Siemens Healthineers SOMATOM go. Currently, the CT scanner is in the supine position at full inspiration. The main acquisition parameters, as recorded in the DICOM headers and summarized in ct_protocol.csv, were as follows:

• Tube voltage: 110 kVp (fixed).
• Slice thickness: 0.8 mm (fixed).
• Reconstruction kernel: Br60-type lung kernel.
• Tube current: mean ≈ 206 mA; range ≈ 75–262 mA.
• Exposure time: mean ≈ 534 ms.
• CTDIvol: mean ≈ 8.96 mGy; range ≈ 3.25–12.52 mGy.

Only the target thin-slice native chest series was used for further processing.

Data extraction includes the following stages: (i) DICOM-NIfTI conversion, (ii) segmentation, and (iii) feature extraction. In parallel, acquisition-level data are obtained and stored in a ct_protocol file. The extraction protocol is depicted in Fig. 1.

### 4.3 DICOM–NIfTI conversion

For each subject, the selected DICOM series was converted to the NIfTI format via dcm2niix (version v1.0.20220720), preserving the voxel spacing and orientation. The resulting volumetric image was used as input for segmentation.

### 4.4 Segmentation workflow

Segmentation was performed via TotalSegmentator (version 2.11.0) [11] with the following logic:

1. Lobe segmentation.
2. Vessel and airway segmentation.
3. Parenchyma mask construction - For each lobe, a parenchymal mask was created by subtracting the union of vessel and airway masks (lobe_parenchyma = lobe_mask − (lung_vessels ∪ lung_trachea_bronchia; Fig. 2).

**Fig. 2:**
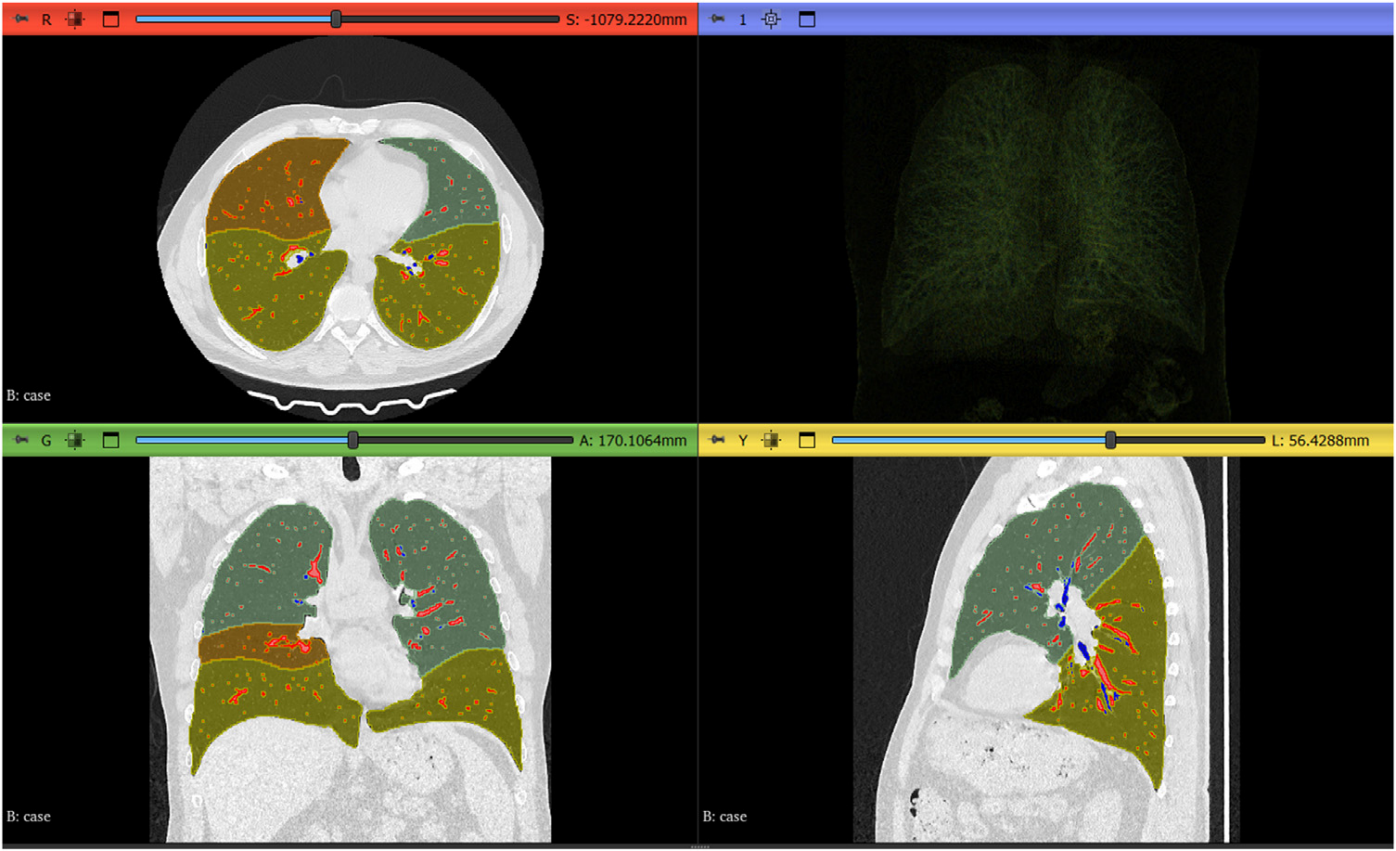
Representative segmentation outputs of the anatomical ROIs. Lobar masks (upper lobes – dark green, lower lobes – olive, middle right – bronze) and intrapulmonary vessel (red)/central airway (blue) masks were generated with TotalSegmentator and visualized in 3D Slicer software. The upper-left, lower-left, and lower-right panels show CT images in the axial, coronal, and sagittal planes, respectively. The upper-right panel shows a 3D reconstruction (volume rendering) of the lung parenchyma.
4. The combined “raw” and “parenchymal” lobes were combined into the left and right lungs, both of which were lungs.

All masks (“raw” and parenchyma lobe- and lung-level, both lungs – total of 16) were stored in NIfTI format during processing and then used as inputs for radiomics extraction.

### 4.5 Radiomics features extraction

Radiomic features were extracted via a CUDA-accelerated fork of PyRadiomics (PyRadiomics-cuda, distribution version 1.0.4; git commit aa3dc733b) on the basis of the original PyRadiomics framework [1] with a fixed configuration (provided in the Python script in the dataset) across all subjects, ROIs, and windows. The key parameters were as follows:

• Interpolation: B-spline interpolation (sitkBSpline).
• Resampled voxel spacing: 1 × 1 × 1 mm.
• Intensity normalization: disabled.
• Resegmentation mode: absolute.
• Resegmentation ranges (Hounsfield units): RAW_-1000_200 (−1000 to +200 HU) and W_-950_0 (−950 to 0 HU).
• Bin width: 25 HU.
• Minimum ROI dimensions: 3 voxels.
• Mask correction: enabled.
• GLCM distances: [1].
• Symmetrical GLCM: True.
• C-extensions: enabled.

For each combination of SubjectID, ROI, and HUWindow, PyRadiomics computes 107 features from the following classes:

• Shape (e.g., volume, surface area, sphericity).
• First-order statistics (e.g., mean, median, variance, skewness, kurtosis, entropy).
• Texture: GLCM, GLRLM, GLSZM, GLDM, NGTDM.

## Limitations

The DICOM files and segmentation masks are not included because Ethics Committee approval does not permit distribution of the source imaging data or derived masks; only deidentified radiomic feature tables and nonidentifying acquisition metadata are shared. Consequently, alternative segmentation approaches, image preprocessing, or independent feature reextraction from the original images cannot be performed via this release. In addition, all CT examinations were acquired at a single center on a single scanner with a uniform protocol, which improves internal consistency but limits transferability to other scanners or reconstruction settings; cross-site reuse may require harmonization.

## Ethics Statement

This study was approved by the Ethical Committee of Ivano-Frankivsk National Medical University, under protocol number 154/25, dated 22.10.2025. The study was conducted in accordance with the Declaration of Helsinki and relevant national regulations, ensuring the confidentiality and integrity of patient data.

Because all the data shared in this dataset were fully deidentified and limited to derived radiomic features and nonidentifiable acquisition parameters, the need to obtain informed consent was waived.

## CRediT Author Statement

**Viktoria Bedei:** Conceptualization; Methodology; Investigation; Formal analysis; Data curation; Writing - Original draft. **Mykola Ostrovskyy:** Conceptualization; Methodology; Investigation; Writing - Original Draft. **Nilanjan Dey:** Validation; Formal analysis; Visualization; Writing - Original Draft; Writing - Review & Editing. **Taras Kotyk:** Software; Formal analysis; Visualization; Writing - Original Draft; Writing - Review & Editing. **R. Simon Sherratt:** Validation; Formal analysis; Writing - Original draft; Writing - Review & editing. Final approval was provided by all the authors.

## Data Availability

Mendeley DataLung CT Radiomics Dataset of Clinically Healthy Adults (Original data)

## References

[1] van Griethuysen J.J.M., Fedorov A., Parmar C., Hosny A., Aucoin N., Narayan V., Beets-Tan R.G.H., Fillion-Robin J.-C., Pieper S., Aerts H.J.W.L. (2017). Computational radiomics system to decode the radiographic phenotype. Cancer Res..

[2] Zwanenburg A., Vallières M., Abdalah M.A., Aerts H.J.W.L., Andrearczyk V., Apte A., Ashrafinia S., Bakas S., Beukinga R.J., Boellaard R., Bogowicz M., Boldrini L., Buvat I., Cook G.J.R., Davatzikos C., Depeursinge A., Desseroit M.-C., Dinapoli N., Dinh C.V., Echegaray S., El Naqa I., Fedorov A.Y., Gatta R., Gillies R.J., Goh V., Götz M., Guckenberger M., Ha S.M., Hatt M., Isensee F., Lambin P., Leger S., Leijenaar R.T.H., Lenkowicz J., Lippert F., Losnegård A., Maier-Hein K.H., Morin O., Müller H., Napel S., Nioche C., Orlhac F., Pati S., Pfaehler E.A.G., Rahmim A., Rao A.U.K., Scherer J., Siddique M.M., Sijtsema N.M., Socarras Fernandez J., Spezi E., Steenbakkers R.J.H.M., Tanadini-Lang S., Thorwarth D., Troost E.G.C., Upadhaya T., Valentini V., van Dijk L.V., van Griethuysen J., van Velden F.H.P., Whybra P., Richter C., Löck S. (2020). The image biomarker standardization initiative: standardized quantitative radiomics for high-throughput image-based phenotyping. Radiology.

[3] Schöneck M., Lennartz S., Zopfs D., Sonnabend K., Wawer Matos Reimer R., Rinneburger M., Graffe J., Persigehl T., Hentschke C., Baeßler B., Lourenco Caldeira L., Hokamp N.Große (2024). Robustness of radiomic features in healthy abdominal parenchyma of patients with repeated examinations on dual-layer dual-energy CT. Eur. J. Radiol..

[4] Armato S.G., McLennan G., Bidaut L., McNitt-Gray M.F., Meyer C.R., Reeves A.P., Zhao B., Aberle D.R., Henschke C.I., Hoffman E.A., Kazerooni E.A., MacMahon H., van Beek E.J.R., Yankelevitz D., Biancardi A.M., Bland P.H., Brown M.S., Engelmann R.M., Laderach G.E., Max D., Pais R.C., Qing D.P.-Y., Roberts R.Y., Smith A.R., Starkey A., Batra P., Caligiuri P., Farooqi A., Gladish G.W., Jude C.M., Munden R.F., Petkovska I., Quint L.E., Schwartz L.H., Sundaram B., Dodd L.E., Fenimore C., Gur D., Petrick N., Freymann J., Kirby J., Hughes B., Vande Casteele A., Gupte S., Sallam M., Heath M.D., Kuhn M.H., Dharaiya E., Burns R., Fryd D.S., Salganicoff M., Anand V., Shreter U., Vastagh S., Croft B.Y., Clarke L.P. (2011). The lung image database consortium (LIDC) and image database resource initiative (IDRI): a completed reference database of lung nodules on CT scans. Med. Phys..

[5] Prior F., Smith K., Sharma A., Kirby J., Tarbox L., Clark K., Bennett W., Nolan T., Freymann J. (2017). The public cancer radiology imaging collections of the cancer imaging archive. Sci. Data.

[6] Colak E., Kitamura F.C., Hobbs S.B., Wu C.C., Lungren M.P., Prevedello L.M., Kalpathy-Cramer J., Ball R.L., Shih G., Stein A., Halabi S.S., Altinmakas E., Law M., Kumar P., Manzalawi K.A., Nelson Rubio D.C., Sechrist J.W., Germaine P., Lopez E.C., Amerio T., Gupta P., Jain M., Kay F.U., Lin C.T., Sen S., Revels J.W., Brussaard C.C., Mongan J., Abdala N., Bearce B., Carrete H., Dogan H., Huang S.-C., Crivellaro P., Dincler S., Kavnoudias H., Lee R., Lin H.-M., Salehinejad H., Samorodova O., Rodrigues dos Santos E., Seah J., Zia A., Arteaga V.A., Batra K., Castelli von Atzingen A., Chacko A., DiDomenico P.B., Gill R.R., Hafez M.A., John S., Karl R.L., Kanne J.P., Mathilakath Nair R.V., McDermott S., Mittal P.K., Mumbower A., Lee C., Orausclio P.J., Palacio D., Pozzessere C., Rajiah P., Ramos O.A., Rodriguez S., Shaaban M.N., Shah P.N., Son H., Sonavane S.K., Spieler B., Tsai E., Vásquez A., Vijayakumar D., Wali P.P., Wand A., Zamora Endara G.E. (2021). The RSNA pulmonary embolism CT dataset. Radiol. Artif. Intell..

[7] Afshar P., Heidarian S., Enshaei N., Naderkhani F., Rafiee M.J., Oikonomou A., Fard F.B., Samimi K., Plataniotis K.N., Mohammadi A. (2021). COVID-CT-MD, COVID-19 computed tomography scan dataset applicable in machine learning and deep learning. Sci. Data.

[8] Han Z., Zhang Y., Ding W., Zhao X., Jia B., Liu T., Wan L., Xing Z. (2025). An integrated mycobacterial CT imaging dataset with multispecies information. Sci. Data.

[9] Bakr S., Gevaert O., Echegaray S., Ayers K., Zhou M., Shafiq M., Zheng H., Benson J.A., Zhang W., Leung A.N.C., Kadoch M., Hoang C.D., Shrager J., Quon A., Rubin D.L., Plevritis S.K., Napel S. (2018). A radiogenomic dataset of non-small cell lung cancer. Sci. Data.

[10] Zhao B., Dercle L., Yang H., Riely G.J., Kris M.G., Schwartz L.H. (2024). Annotated test-retest dataset of lung cancer CT scan images reconstructed at multiple imaging parameters. Sci. Data.

[11] Wasserthal J., Breit H.-C., Meyer M.T., Pradella M., Hinck D., Sauter A.W., Heye T., Boll D.T., Cyriac J., Yang S., Bach M., Segeroth M. (2023). Total segmentator: robust segmentation of 104 anatomic structures in CT images. Radiol. Artif. Intell..

[12] Zhao M., Wu Y., Li Y., Zhang X., Xia S., Xu J., Chen R., Liang Z., Qi S. (2024). Learning and depicting lobe-based radiomics feature for COPD Severity staging in low-dose CT images. BMC Pulm. Med..

[13] Chatterjee A., Valliéres M., Forghani R., Seuntjens J. (2021). Investigating the impact of the CT Hounsfield unit range on radiomic feature stability using dual energy CT data. Phys. Med..

[14] Joye A.A., Bogowicz M., Gote-Schniering J., Frauenfelder T., Guckenberger M., Maurer B., Tanadini-Lang S., Gabryś H.S. (2024). Radiomics on slice-reduced versus full-chest computed tomography for diagnosis and staging of interstitial lung disease in systemic sclerosis: a comparative analysis. Eur. J. Radiol. Open.

